# Essential Oils and Their Major Compounds in the Treatment of Chronic Inflammation: A Review of Antioxidant Potential in Preclinical Studies and Molecular Mechanisms

**DOI:** 10.1155/2018/6468593

**Published:** 2018-12-23

**Authors:** Érica Martins de Lavor, Antônio Wilton Cavalcante Fernandes, Roxana Braga de Andrade Teles, Ana Ediléia Barbosa Pereira Leal, Raimundo Gonçalves de Oliveira Júnior, Mariana Gama e Silva, Ana Paula de Oliveira, Juliane Cabral Silva, Maria Tais de Moura Fontes Araújo, Henrique Douglas Melo Coutinho, Irwin Rose Alencar de Menezes, Laurent Picot, Jackson Roberto Guedes da Silva Almeida

**Affiliations:** ^1^Center for Studies and Research of Medicinal Plants, Federal University of San Francisco Valley, 56304-205 Petrolina, Pernambuco, Brazil; ^2^UMRi CNRS 7266 LIENSs, University of La Rochelle, La Rochelle, France; ^3^Department of Biological Chemistry, Regional University of Cariri, 63105-000 Crato, Ceará, Brazil

## Abstract

Inflammatory diseases result from the body's response to tissue damage, and if the resolution is not adequate or the stimulus persists, there will be progression from acute inflammation to chronic inflammation, leading to the development of cancer and neurodegenerative and autoimmune diseases. Due to the complexity of events that occur in inflammation associated with the adverse effects of drugs used in clinical practice, it is necessary to search for new biologically active compounds with anti-inflammatory activity. Among natural products, essential oils (EOs) present promising results in preclinical studies, with action in the main mechanisms involved in the pathology of inflammation. The present systematic review summarizes the pharmacological effects of EOs and their compounds in *in vitro* and *in vivo* models for inflammation. The research was conducted in the following databases: PubMed, Scopus, BIREME, Scielo, Open Grey, and Science Direct. Based on the inclusion criteria, 30 articles were selected and discussed in this review. The studies listed revealed a potential activity of EOs and their compounds for the treatment of inflammatory diseases, especially in chronic inflammatory conditions, with the main mechanism involving reduction of reactive oxygen and nitrogen species associated with an elevation of antioxidant enzymes as well as the reduction of the nuclear factor kappa B (NF-*κ*B), reducing the expression of proinflammatory cytokines. Thus, this review suggests that EOs and their major compounds are promising tools for the treatment of chronic inflammation.

## 1. Introduction

Inflammation is characterized as a normal response to tissue damage caused by several potentially injurious stimuli, induced by biological, chemical, and physical factors [1]. Initially, inflammatory agents elicit an acute inflammatory response which generally promotes complete destruction of the irritants. This type of inflammation persists for a short time and is beneficial for the host [2, 3]. However, if resolution of inflammation is inadequate or the stimulus persists, chronic inflammation occurs, predisposing the host to various diseases including, for example, cancer and neurodegenerative diseases [4–6].

During chronic inflammation, a variety of intracellular signaling pathways are activated, comprising of cell surface receptors, tyrosine kinases, and transcription factors, leading to overexpression of proinflammatory genes involved in the development of chronic diseases [2]. Furthermore, the cellular components represented by the mast cells and leukocytes are recruited to the site of the damage, which leads to a “respiratory burst” result of increased oxygen uptake and therefore an increased release and accumulation of reactive oxygen species (ROS) and reactive nitrogen species (RNS) at the site of damage [2, 4–7]. Under physiological conditions, ROS are generated in phagocytes to neutralise the invading organisms, presenting an important role in the host defense mechanism. In contrast to oxidant mechanisms, the organism has endogenous defense antioxidant systems, including for example superoxide dismutase, glutathione peroxidase, and catalase. When ROS production is greater than cellular antioxidant capacity, oxidative stress can damage DNA, proteins, and lipids [8–11].

A diversity of protein kinases is activated in the inflammatory process, such as members of the Janus-activated kinase (JAK), phosphatidylinositol-3-kinase (PI3K/, AKT), and mitogen-activated protein kinase (MAPK) families to alter cell proliferation. In the chronic inflammatory process, the excessive activation of these signaling pathways causes also the activation of certain transcription factors, such as NF-*κ*B, signal transducer and activator of transcription 3 (STAT3), hypoxia-inducible factor-1*α* (HIF1-*α*), and activator protein-1 (AP-1), potentiating the initial inflammatory response. In addition to these factors, the cyclooxygenase enzyme (COX), inducible nitric oxide synthase (iNOS), cytokines, and chemokines have also been reported to play a role in oxidative stress-induced inflammation [2, 12, 13].

In recent years, the search for more effective drugs for the treatment of the inflammation with fewer side effects has encouraged researchers to study and develop new drugs. The search for natural products derived from plants is a promising reality, and among the substances with pharmacological potential we can cite the essential oils (EOs).

EOs are liquid mixtures of volatile compounds obtained from aromatic plants, which represent a small fraction of the plant composition [14]. However, they are responsible for providing characteristics that favor their use in the food, cosmetic, and pharmaceutical applications. Essential oils have a complex composition; the great majority of the identified components include terpenes (oxygenated or not), predominantly monoterpenes and sesquiterpenes. However, allyl and propenylphenols (phenylpropanoids) also are important components of some essential oils [15–17]. These secondary metabolites have been related as potent antioxidants, free radical scavengers, and metal chelators, also presenting antinociceptive, neuroprotective, anticonvulsant, and anti-inflammatory properties, reported in preclinical studies, characterizing as potential source for the development of new drugs [17–20].

The objective of this review was to relate the use of essential oils correlating its antioxidant effect in the treatment of chronic inflammations.

## 2. Material and Methods

### 2.1. Search Strategy

In this review, the specialized databases PubMed, Science Direct, Scopus, Open Grey, Scielo, and BIREME were used for literature search in March and April 2018, using different combinations of the following keywords: essential oils, volatile oils, antioxidants, and inflammation. We did not contact investigators, and we have not attempted to identify unpublished data until the date of the search.

### 2.2. Study Selection

In this step, two independent researchers (J.C.S. and A.W.C.F.) first selected the articles according to title and abstract and finally through an analysis of the full-text publication. The following inclusion criteria were applied: studies with EOs or their major compounds with anti-inflammatory and antioxidant activity *in vitro* and/or *in vivo*. Studies were excluded according to the following exclusion criteria: review articles, meta-analyses, abstracts, conference proceedings, editorials/letters, case reports and studies in humans, and articles published over 20 years ago. Additional papers were included in our study after analyses of all references from the selected articles. In cases of nonconsensus, a third independent review was consulted (E.M.L.) for final decision.

### 2.3. Data Extraction

Data were collected and examined by one reviewer using standardized forms and were checked by a second reviewer. The information extracted from the articles included EOs or their major compounds, cell lines (*in vitro* studies), animal models (*in vivo* studies), doses or concentrations, routes of administration, biochemical assays, and molecular mechanisms investigated.

### 2.4. Methodological Quality Assessment

The risk of bias and quality of preclinical *in vivo* studies were performed using an adapted checklist [21–23]. This investigation allowed assessing the methodological quality of the included studies concerning mainly the randomization of the treatment allocation, blinded drug administration, blinded outcome assessment, and outcome measurements.

## 3. Results and Discussion

### 3.1. Study Selection

**Figure 1:**
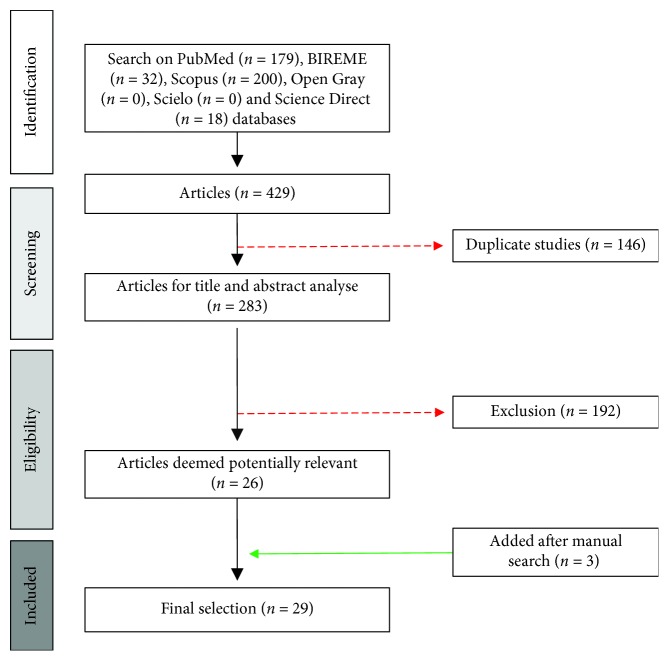
Flowchart detailing literature searching and screening.

The primary search identified 429 articles (200 from Scopus, 18 from Science Direct, 32 from BIREME, and 179 from PubMed). However, 146 manuscripts were indexed in two or more databases and considered only once, resulting in 283 original articles. After an initial screening of titles and abstracts, 192 articles were excluded because they did not meet the inclusion criteria or presented completely different themes from the proposal of this review. After an initial screening of titles and abstracts and a full-text analysis, 27 articles were considered potentially relevant. In addition, 3 articles were included after manual search for data extraction, totalizing 30 final articles included in this systematic review. A flowchart illustrating the progressive study selection and numbers at each stage is shown in Figure 1.

### 3.2. Characteristics of Included Studies

The selected final articles were carefully analyzed in relation to the country where the study was conducted, year of publication, family of the studied species, and whether the study was carried out with essential oils or substances obtained from them.  Table 1 summarizes general information contained in the selected *in vitro* and *in vivo* studies.

Studies were conducted by research groups located in about 13 different countries. Most of the investigations were authored by researchers from Brazil (7 reports, 24.13%), China (6 reports, 20.68%), and India (5 reports, 17.24%).

The largest number of studies found in Brazil is justified by the fact that Brazil has an extremely rich biodiversity, corresponding to approximately 20% of all living species known globally, comprising over 45,000 species of higher plants. In addition, the Brazilian population has a historical tradition in the use of medicinal plants for the treatment of different diseases, including acute and/or chronic inflammation disorders [54, 55]. Another interesting fact is the number of studies conducted in China and India, which may be justified as a reflection of the contribution of Oriental medicine in the search and development for new drugs from natural products. In fact, traditional Chinese medicine (TCM) and Ayurveda as major traditional treatment systems used not only in India and China but also in several countries contributed to the development of new pharmaceutical products based on plant species [56–58].

**Figure 2:**
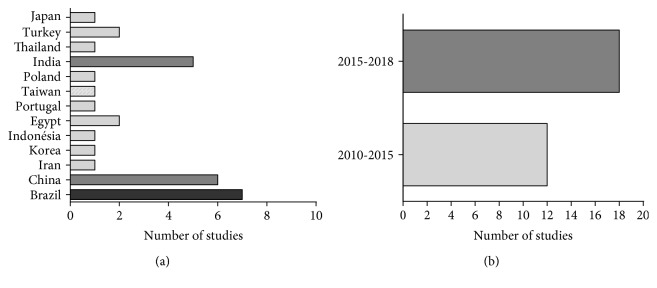
Distribution of the selected studies by country (a) and year of publication (b).

Regarding the number of annual publications, we noted that a large number of articles were published from 2010 to 2015 (12 reports). Only in the last three years were 18 studies (62.02%) found, suggesting that the involvement of oxidative stress in anti-inflammatory activity of essential oils or their major compounds has attracted the attention of the researchers in diverse regions of the world. These results are graphically presented in Figure 2.

Among the included articles, only 10 (32.25%) corresponded to studies with isolated components of essential oils, demonstrating that reports involving EOs are still predominant in this subject. Of these oils, three studies were reported for species belonging to the Rutaceae family and two studies for the families Zingiberaceae, Apiaceae, Cupressaceae, and Lamiaceae. The other studies correspond to other families reported in Table 1.

As described in Table 1, our review included 2 reports presenting in *vitro* and *in vivo* studies, 9 reports presenting only *in vitro* studies, and 19 reports presenting only *in vivo* studies. In the studies reported in this review, biochemical and molecular targets were verified by colorimetric and enzymatic assays, biochemical analyses, and techniques such as Western blot and immunohistochemistry. These studies base their assays on methodologies using cell culture commonly found in chronic inflammatory processes, such as macrophages, monocytes, astrocytes, and cancer cells, correlating anti-inflammatory results with the antioxidant potential of essential oil or their major components. The evaluated *in vitro* studies parameters and main outcomes are summarized in Table 2 and *in vivo* studies in Table 3.

### 3.3. Methodological Quality of In Vivo Studies

**Figure 3:**
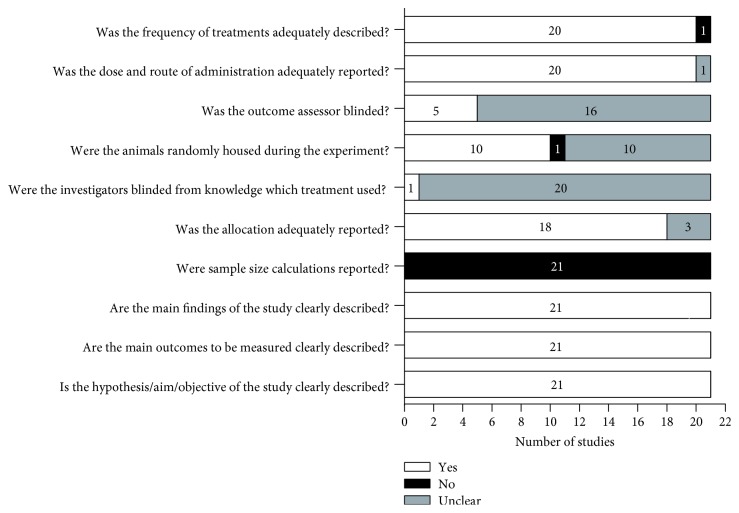
Methodological quality of included *in vivo* studies. White bars indicate the proportion of articles that met each criterion; black bars indicate the proportion of studies that did not, and gray bars indicate the proportion of studies with unclear answers.

Regarding methodological quality, all *in vivo* studies were evaluated using a standardized checklist, as shown in Figure 3. It was observed that all studies described the objectives, outcomes to be measured, and main findings obtained. However, none of the included articles reported sample size calculation. In general, doses, routes of administration, and frequency of treatment were adequately described. Most of the *in vivo* studies (18 reports, 85.7%) adequately reported the animal allocation, but less than half (10 reports, 47.6%) reported that the animals were randomly housed. In addition, the majority of included studies did not make it clear if the investigators or the outcome assessor were blinded from the treatment used.

The number of animals to be used, randomization, and blinding are important steps in preclinical protocols in order to reduce the risk of bias and improve translatability of animal research [59, 60]. In this way, the evaluation of the methodological quality indicated that the majority of *in vivo* studies included in this review present moderate quality, which limits the interpretation of the results.

### 3.4. In Vitro Tests of Anti-inflammatory Activity

Researchers, when proposing to investigate the pharmacological evaluation of substances, initially carry out *in vitro* tests, since these tests present a high reproducibility and function as a trait to evaluate the pharmacological potential of these substances, as for example the anti-inflammatory activity. The assays employed are, in most instances, cell culture techniques, in which the cells receive various stimuli (chemical or biological) that induce the production of mediators involved in inflammatory processes, such as arachidonic acid and cytokines and their metabolites [24, 28, 30, 42].

In the majority of *in vitro* selected papers, the anti-inflammatory activity tests employed the macrophage cell line RAW 264.7 activated by LPS [28, 30, 41, 42, 48]. Macrophages play a critical role in the inflammatory process through the production of various cytokines. When these cells are activated, they express the inflammatory enzymes (iNOS and COX-2) and proinflammatory cytokines (TNF-*α* and IL-6). However, they also may play an anti-inflammatory role in which they express IL-4, IL-13, or IL-10 cytokines [61–63].

Other cells participate in the inflammatory process and have a crucial role in the development of inflammatory diseases. To evaluate this activity, Singh et al. [64] proposed the utilization of the human THP-1 cell, a common model to estimate modulation of monocyte and macrophage activities. Circulating monocytes have the potential to differentiate into tissue macrophages, providing help in the phagocytosis of invading pathogens, reducing tissue aggression by potentially harmful agents [65].

In recent years, inflammatory processes have been correlated to the development of chronic diseases. However, chronic inflammation and cytokine dysfunction are associated with conditions such as cancer progression, cardiovascular disease, diabetes, and neurodegenerative disease [66]. To better study these molecular aspects, inflammatory models using microglial [29] and astrocyte [43] cell lines have been used to evaluate the influence of inflammatory processes on the development of neurodegenerative diseases and tumor cell lines such as HepG2 [27] to evaluate the relationship between the processes inflammatory and malignant neoplasms.

### 3.5. Animal Models in Chronic Inflammation

Chronic inflammation is an aggravating factor for tissue damage, commonly present in many chronic diseases, including asthma, obstructive pulmonary disease, and neuroinflammatory and autoimmune disorders [67]. For this reason, it is necessary to understand the molecular mechanisms involved in the inflammatory process in order to develop new treatment and prevention protocols. Thus, many experimental models have been developed, most often using mice and rats, in order to correlate the pathophysiology of the disease and to aid in the development of new drugs [68, 69].

Concerning *in vivo* studies included in this review, EOs were investigated in experimental models of ulcerative enterocolitis; lesions developed by chemotherapeutic agents; peritoneal, subcutaneous, pulmonary, and cardiac inflammation induced by biological and chemical agents; and atherosclerosis.

In recent years, some reports relate pathogen infection to the development and progression of chronic inflammation. In this systematic review, we found 9 studies reporting inflammatory conditions induced by microorganisms or their components, including LPS from *E. coli*, complete Freund's adjuvant, *S. flexneri*, and *P. multocida* [22, 31, 34, 36, 39, 40, 44, 50]. LPS is the major cause of endotoxemia and sepsis. Toll-like receptor 4 (TLR4) is the main way to activate the inflammation pathway. Some authors suggest that LPS would stimulate inflammation by stimulating the production of reactive oxygen species, mainly by the production of superoxide anion (O_2_^−^); these factors activate for example the MAPK pathway that will trigger cellular responses to increase the production of proinflammatory cytokines to evoke the immune system to fight the injury [44, 70].

In addition, administration of LPS or microorganisms induces transcription factor NF-*κ*B for initiating and sustaining inflammatory reactions. In the cell cytoplasm, NF-*κ*B is inactivated by the interaction with newly synthesized protein inhibitory *κ*B (I*κ*B), in the TLR4 signaling-promoted dissociation of complex I*κ*B-NF-*κ*B and translocation of NF-*κ*B into the nucleus from the cytoplasm to induce gene transcription of cytokines and chemokines [71].

Another widely used model corresponds to the evaluation of inflammatory bowel disease (IBD), for which several pharmacological models are employed, such as induction of ulcers by *S. flexneri* strains, intestinal mucositis induced by chemotherapy, and enterocolitis induced by DMH [40, 47, 51]. These pathologies are characterized by an excessive response of the immune system of the intestinal mucosa, activating the production and release of inflammatory mediators, such as eicosanoids, cytokines, reactive oxygen species (ROS), and nitrogen. In addition, defense cells such as mast cells produce toxic superoxide anions in the inflammatory environment and recruit neutrophils generating excess ROS, proteolytic enzymes, and ROS that contribute to lipid peroxidation. Furthermore, activated macrophages, neutrophils, and mast cells express receptors for IL-1*β* and iNOS playing an important role in progression or persistence of intestinal lesion [72–74].

Involvement of inflammation in the pathogenesis of atherosclerosis is also well documented. Inflammatory cell types such as T-cells, monocytes, and neutrophils play major roles in mediating the inflammatory response in atherosclerosis. The deposition of lipid and oxidized low-density lipoprotein contributes to the initial and prolongated inflammatory response, especially in lipid oxidation, which is taken up by macrophages, dendritic cells, and smooth muscle cells to form lipid-laden foam cells. In addition, cells of the immune system participate to the inflammatory process producing proinflammatory cytokines IL-1 and TNF-*α*, mediators associated with reactive oxygen species- (ROS-) and nitric oxide- (NO-) (in excess) induced expression of adhesion molecules, and potentiate inflammation within the atherosclerotic lesion, which induces the chemoattraction of defense cells [49, 75–77].

Other experimental models have been well reported to assess chronic inflammation, such as cotton-pellet-induced granuloma, subcutaneous air pouch, and formalin test. However, these tests present low similarity to the previously described models in relation to the ability to resemble specific human inflammations, since they reproduce the general aspects of the chronic inflammatory process [26, 33, 35, 38].

The formalin test is commonly described in acute inflammation tests; however, repeated application was described in the studies of Jeena et al. [26, 35]. The inflammatory process is a result of tissue and functional alterations in the tissue accompanied by the release of inflammatory mediators such as histamine, prostaglandins, nitric oxide, and cytokines. To evaluate this, the authors monitor the reduction of edema and perform dosage of the involved mediators [26, 35, 78, 79].

The granulomatous tissue induced by the subcutaneous cotton implant is a widely used method for the assessment of anti-inflammatory substance in chronic inflammation. This type of inflammation is a result of several infectious, autoimmune, toxic, allergic, and neoplastic conditions, characterized by the presence of mononuclear leukocytes, specifically macrophages, which respond to several chemical mediators of cell damage, most often forming multinucleated giant cells. In the injured tissue, some histological patterns are observed, such as edema, neovascularization, and early-stage fibrosis [80, 81].

### 3.6. Role of Antioxidants in Chronic Inflammation

Free radicals correspond to a molecule or atom that carries unpaired electrons that makes them highly reactive and unstable and can cause cell damage. In normal cell metabolism, many free radicals are produced, which serve important functions in the signaling of specific pathophysiological pathways, the great majority of these radicals being produced in the mitochondrial metabolism. Examples of these are hydroxyl radical, superoxide anion, hydrogen peroxide, and organic peroxides. In addition, in the absence or low concentrations of oxygen, excessive lipid peroxidation occurs and mitochondria also generate nitric oxide (NO), which can generate reactive nitrogen species, which can produce other reactive species such as malondialdehyde [11, 82, 83].

In the inflammatory process, defensive cells located in injured regions lead to a “respiratory burst” in the tissue resulting from increased uptake of oxygen and, therefore, increased production and release of ROS in the damaged area. The release of mediators by these cells associated with the presence of ROS and RNS stimulates signal transduction cascades and alters transcription factors, such as NF-*κ*B, which mediate vital reactions of cellular stress, leading to expression of COX-2, iNOS, and proinflammatory cytokines. Metabolites generated in inflammation associated with oxidative stress impair healthy tissue by altering the stroma and surrounding epithelial cells, which after a long period of time can evolve into more serious problems and trigger, for example, carcinogenesis [84, 85].

In general, the body has an enzymatic system to combat the damage caused by oxidative stress. Three major antioxidants are the first line of defense against oxidative stress: superoxide dismutase, catalase, and glutathione peroxidase, being antioxidants commonly measured in the investigation of the antioxidant activity of natural compounds [86, 87].

SOD enzyme, which converts highly reactive superoxide radicals in hydrogen peroxide (H_2_O_2_) and molecular oxygen [86], performs a first antioxidant defense in an oxidative stress situation [88]. Catalase also participates in this defense process, catalyzing the conversion of hydrogen peroxide (highly reactive) to water and molecular oxygen, being located mainly in the peroxisomes [89]. Glutathione exists in two forms with different subunits and different active sites. Glutathione peroxidase catalyzes the reduction of H_2_O_2_ or organic peroxides (ROOH) to water or alcohol by the presence of GSH, which is converted to oxidize glutathione during this reaction. The main function of this latter enzyme is the protection of the polyunsaturated cell membranes [87].

In the articles reported in this study, the authors correlated the antioxidant tests with the anti-inflammatory activity of the essential oils and substances tested. For this, isolated tests of *in vitro* antioxidant activity, such as DPPH, inhibition of *β*-carotene degradation, ABTS, NO, and FRAP tests, were used as initial screening of the pharmacological activity of EOs or isolated substances. Based on the satisfactory results obtained, some anti-inflammatory activity tests were subsequently conducted [24, 27, 29–31, 40, 48, 50]. In contrast, other studies, especially *in vivo* studies, assessed the anti-inflammatory activity and at the end of the experiment the animals were euthanized and blood collected for serum levels indicating enzymes of oxidative stress. In these models, the analyses of superoxide dismutase, catalase, glutathione, malondialdehyde, and lipid peroxidation were mostly described [25, 26, 32, 35, 36, 39, 46, 47, 49].

### 3.7. Essential Oils with Antioxidant Properties in the Treatment of Chronic Inflammation

**Figure 4:**
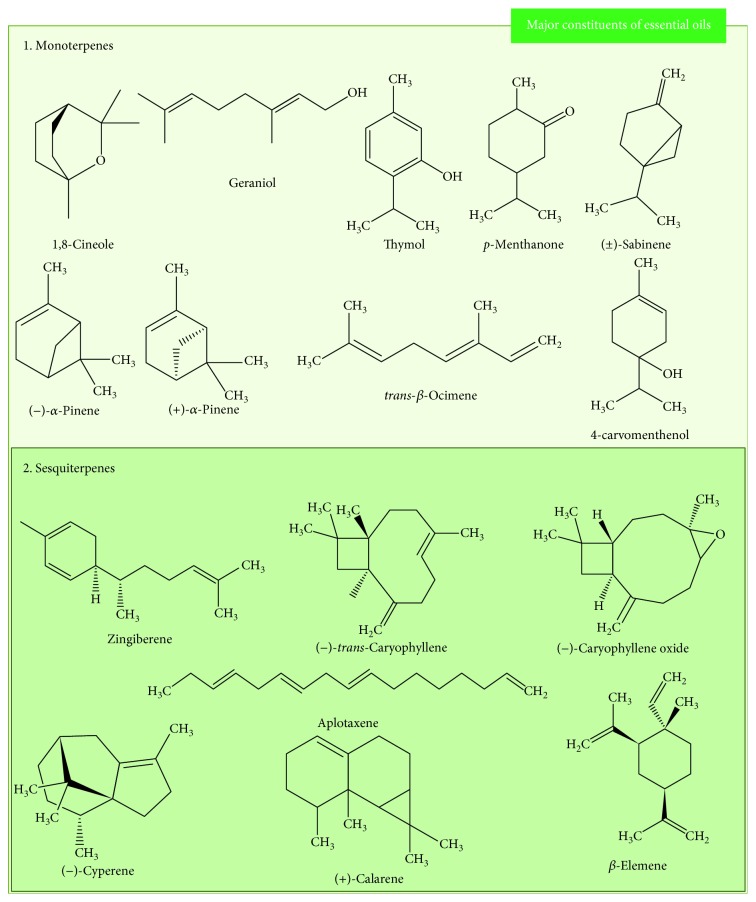
Chemical structure of the major constituents of the essential oils evaluated as antioxidant and anti-inflammatory in chronic inflammation.

**Figure 5:**
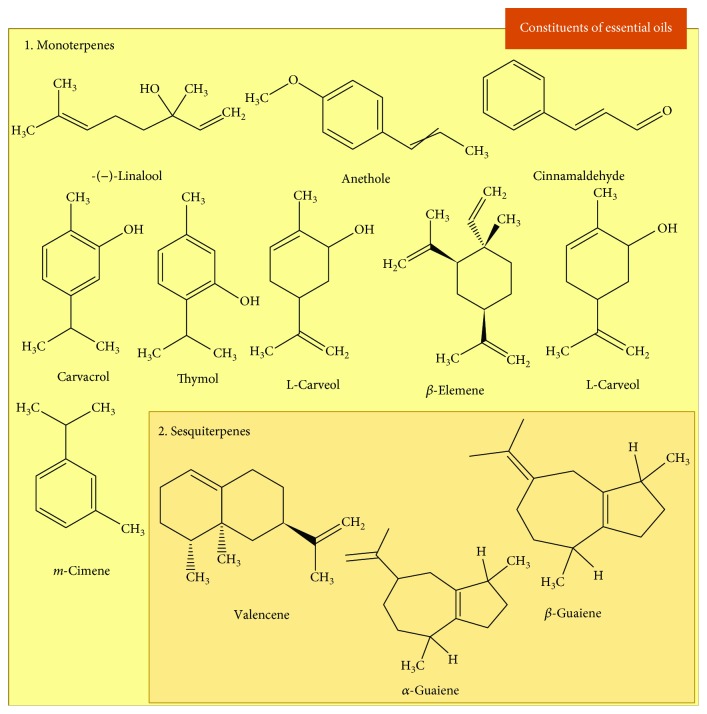
Chemical structure of the constituents isolated of the essential oils evaluated as antioxidant and anti-inflammatory in chronic inflammation.

In view of the wide use of traditional medicine associated with its importance in drug discovery, EOs have been studied and their compounds identified/isolated components due to their diverse pharmacological properties, including the treatment of acute and chronic inflammation justified by their antioxidant properties [16, 17]. EOs are volatile compounds that may contain more than 300 different compounds. Most of chemical constituents are terpenes, especially mono- and sesquiterpenes, but some nonterpene compounds biosynthesized by the phenylpropanoid pathway can also be present in EOs [14].  Figure 4 shows the major constituents of the EOs reported in this study, and Figure 5 shows the isolated constituents with antioxidant activity tested in chronic inflammation models.

The anti-inflammatory and antioxidant activities of species and natural compounds were reported in the studies included in this article, where numerous preclinical cstudies presented promising results. In the experiments using peritoneal macrophages (Raw 264.7, *in vitro*), EOs obtained from *O. crocata*, *O. quixo*, *C. intratropica*, *C. reticulata*, *H. sabdariffa*, *C. obtusa*, and *C. aurantium* were able to reduce the levels of ROS considerably reducing tissue inflammation and RNS that cause tissue damage. In addition, EO of *H. sabdariffa* and *C. aurantium* inhibited NF-*κ*B and MAPK signaling and promoted the decrease in the expression of transcription factors for the production of cytokines IL-1*β*, IL-6, and TNF-*α*. In addition, these EOs decreased the expression of cyclooxygenase-2 and iNOS enzymes [28, 30, 41, 42, 48].

*In vivo* tests indicated that the treatment with ginger and *P. nigrum* EOs presents the ability to sequester superoxide, DPPH, and hydroxyl radicals, in addition to inhibiting lipid peroxidation, associated with the reduction in edema induced by chronic administration of formalin in paw tissue. In this way, the decrease in the inflammatory process occurs due to the increase in the activity of antioxidant enzymes SOD and glutathione [26, 35]. In contrast, *C. aurantium* and *C. ternata* reduced defense cell migration and edema and reduced the levels of nitric oxide in the inflammatory exudate in a granuloma model. In inflammation induced by biological agents such as *E. coli* LPS, treatment with EO of *N. sativa* increased SOD and CAT expression and reduced nitric oxide and malondialdehyde levels [29, 33].

*P. integerrima* presented potential antiasthmatic activity in preclinical studies. This activity is related to inhibition of the degranulation of mast cell and inhibition of 5-LOX, where treatment with EO considerably reduced the number of total leukocytes in bronchoalveolar lavage fluid and pulmonary levels of myeloperoxidase. Associated with this, the plant presented antioxidant potential in the DPPH test, indicating satisfactory results for the treatment of chronic pulmonary diseases with possible involvement of oxidative pathways [31].

Articles that report the pharmacological evaluation of the essential oil of *Z. cassumunar* in encapsulated niosome by therapeutic ultrasound were also found in the searches. The encapsulated niosomes were applied in the skin and subsequently evaluated using ultrasound therapy to potentiate the anti-inflammatory action of the EO, favoring the absorption by the skin and subsequent action on the inflammation induced by repeated administration of LPS. The anti-inflammatory action of this oil is probably related to the presence of sabinene and terpinen-4-ol (major compounds) which reduce the expression of NF-*κ*B and interleukin-6. The antioxidant tests indicated antioxidant activity of the EO, which inhibited DPPH radical, demonstrating once again the relation of inflammatory processes and antioxidant mechanisms [50].

**Figure 6:**
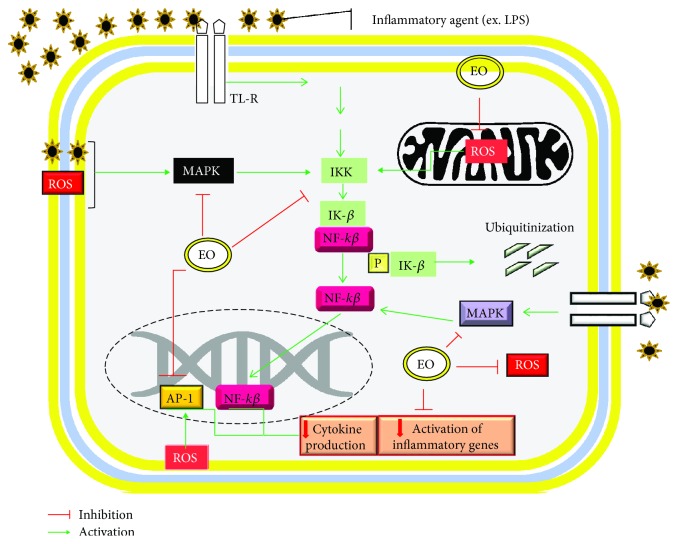
Molecular mechanisms of action of essential oils activity mediating signaling involving inhibition of NF-*κ*B, MAPK, and decreased intracellular oxidative stress.

In relation to the majority compounds studied, most are classified as monoterpenes, such as carvacrol, thymol, L-carveol, L-carvone, and m-cymene (Figure 6).

The carvacrol (5-isopropyl-2-methylphenol) is a phenolic monoterpene present in EOs of various species especially the Lamiaceae family, which presented pharmacological potentials, such as antioxidant and anti-inflammatory [90, 91]. The compound was the most reported in the studies included in this article, exhibiting activity in preclinical models of inflammatory diseases of the gastrointestinal tract, such as chemotherapy-induced mucositis and DMH-induced colitis. Its pharmacological activity in the mentioned models is a result of reduced expression of NF-*κ*B, COX-2, and iNOS, associated with decreased levels of IL-1*β*, TNF-*α*, and NO. The treatment was also able to increase the antioxidant enzymes SOD, CAT, MDA, and GSH [47, 51].

Anethole (1-methoxy-4-benzene-[1-propenyl]) is an aromatic compound used in the industry, which has antioxidant, antibacterial, antifungal, and anti-inflammatory potential [92, 93]. Oral treatment with anethole inhibited complete Freund adjuvant-induced paw edema, in addition to reducing myeloperoxidase levels, TNF-*α*, IL-1*β*, and IL-17, thereby reducing the levels of ROS in the injured tissue [25].

Another terpene described in the articles was linalool (3,7-dimethylocta-1,6-dien-3-ol), which was investigated to assess its ability to reduce *P. multocida*-induced lung inflammation. Repeated subcutaneous administration of linalool reduced the levels of TNF-*α* and IL-6 and the number of polymorphs (neutrophils) in lung tissue, associated with an increase in SOD [34, 94]. Eucalyptol (1,3,3-trimethyl-2-oxabicyclo[2.2.2]octane) was also evaluated in a model of chronic lung inflammation induced by repeated exposure to cigarette smoke, where the treatment reduced the expression of NF-*κ*B and consequently the levels of proinflammatory cytokines, promoting the reduction of the presence of leukocytes in the pulmonary alveoli. The levels of antioxidant enzymes SOD, CAT, MDA, and GSH, as well as total ROS were reduced [46].

In the experimental models, cinnamaldehyde, *β*-elemene, and thymol were evaluated in LPS-induced cardiac inflammation or hyperlipidic diet. Cinnamaldehyde possesses potent anti-inflammation effects on endotoxemia [94]. Zhao et al. [44] showed that cinnamaldehyde inhibited inflammatory infiltration and the levels of TNF-*α*, IL-1*β*, and IL-6 in LPS-stimulated rats by blocking the TLR4 and MAPK pathways, associated with the reduction in ROS levels in cardiac tissue [44]. *β*-Elemene also showed activity in the cardiac inflammation model. In this evaluation, treatment of apolipoprotein E (ApoE) knockout mice with *β*-elemene inhibited atherosclerotic lesions by reducing levels of nitric oxide, cytokines, and oxidative stress indicators and reversing the intracellular ROS production and MAPK signaling activation [49].

Thymol (2-isopropyl-5-methylphenol) was evaluated in two different models, inflammation in aortic intimal and imiquimod-induced psoriasis. In the first model, the antioxidant tests were evaluated *in vitro* DPPH and ABTS radical scavenging assay, demonstrating high antioxidant activity. The treatment also reduced lipid peroxidation *in vivo*, reducing serum levels of malondialdehyde. In relation to the parameters of anti-inflammatory activity, thymol reduced the expression of vascular adhesion molecules (VCAM), thus reducing leukocyte migration and proinflammatory cytokines [45]. Thus, the results suggest that this monoterpene reduced the oxidative stress, the putative mechanism involved in the pathogenesis of endothelial dysfunction, an early key event in the progression of atherosclerosis [95].

Nanoparticles containing thymol were also evaluated, using experimental models that mimic psoriasis. For this, anthralin (1,8-dihydroxy-9-anthrone), a drug used to treat psoriasis, was used for inducing inflammation in healthy skin mice and the antioxidant activity was evaluated after exposition to light, generators, and oxidative stress events. Thymol in nanoparticles showed better inhibition of edema by reducing inflammatory cells in inflamed tissue when compared to free thymol, indicating that nanoparticles improve anti-inflammatory activity mediated by mechanisms that inhibit the formation of reactive oxygen species [53, 96].

In general, the results of the studies indicated that EOs and/or their compounds presented pharmacological properties through the blockade of mitogen-activated protein kinase (MAPK) pathways, blocking NF-*κ*B activation by mechanisms associated with the reduction of oxidative stress, leading to the reduction in the production of several proinflammatory mediators (Figure 6).

## 4. Conclusion and Perspectives

This systematic review suggests that EOs and their major compounds have a potential for the treatment of inflammatory diseases especially in chronic inflammatory conditions. The main action targets presented in this review for the therapy of chronic inflammations were the reduction in reactive oxygen and nitrogen species and the reduction in NF-*κ*B reducing the expression of proinflammatory cytokines.

*In vivo* tests reported various models of inflammation that resemble human pathologies, including assessment of their mechanism of action, antioxidant enzyme dosages, and molecular effects of EOs. Regarding the rigor of design and study data included in this review, most of the studies presented moderate quality indicating that some aspects still need to be improved but in general provide evidence of the anti-inflammatory potential associated with the antioxidant activity of EOs.

## Figures and Tables

**Table 1:** General characteristics of included studies (*in vitro* and *in vivo* reports).

Authors, year, country	Model	Essential oil	Major constituents	Family	Induction of inflammation	Type of inflammation
Tsai et al., 2011, Taiwan [24]	*In vitro*	Essential oils of the aerial parts of *Eucalyptus bridgesiana*, *Cymbopogon martinii*, *Thymus vulgaris*, *Lindernia anagallis*, and *Pelargonium fragrans*	1,8-Cineole Geraniol Thymol *p*-Menthanone (−)-Spathulenol	Myrtaceae Poaceae Lamiaceae Linderniaceae Geraniaceae	Lipopolysaccharide (LPS) from *Escherichia coli* and heat-killed *Propionibacterium acnes*	Inflammation induced by biological agent

Ritter et al., 2013, Brazil [25]	*In vivo*	—	Anethole	—	Complete Freund's adjuvant	Inflammation induced by biological agent

Jeena et al., 2013, India [26]	*In vivo*	Essential oil of ginger	Zingiberene	Zingiberaceae	Formalin	Inflammation induced by chemical agent

El-Readi et al., 2013, Egypt [27]	*In vitro*	Essential oils from leaves and stems of *Liquidambar styraciflua*	*α*-Pinene	Altingiaceae	LPS from *Escherichia coli*	Inflammation induced by biological agent

Valente et al. 2013, Portugal [28]	*In vitro*	Essential oils of the aerial parts of *Oenanthe crocata L.*	*β-*Ocimene Sabinene	Apiaceae	LPS from *Escherichia coli* and INF-*γ*	Inflammation induced by biological agent

Lin et al., 2014, China [29]	*In vitro*	Essential oil of *Patrinia scabiosaefolia*	Caryophyllene oxide	Caprifoliaceae	LPS from *Escherichia coli*	Inflammation induced by biological agent

Destryana et al., 2014, Indonesia [30]	*In vitro*	Essential oil from leaf and branches of *Ocotea quixos*, wood, branches, and leaves of *Callitris intratropica* and *Copaifera reticulata/langsdorffii* gum-resin	*trans*-Caryophyllene *β*-Caryophyllene (+)-Calarene	Lauraceae Cupressaceae Fabaceae	LPS from *Escherichia coli*	Inflammation induced by biological agent

Shirole et al., 2014, India [31]	*In vitro* and *in vivo*	Essential oil of *Pistacia integerrima*	4-Carvomenthenol	Anacardiaceae	LPS from *Escherichia coli* and ovalbumin	Inflammation induced by biological agent

Patil et al. 2014, India [32]	*In vivo*	Essential oil of *Camellia reticulata* L.	—	Theaceae	Indomethacin	Inflammation induced by chemical agent

Khodabakhsh et al. 2014, Japan [33]	*In vivo*	Essential oil from blossoms of *Citrus aurantium* L.	Linalool	Rutaceae	Cotton pellet—subcutaneous	Inflammation induced by physical agent

Wu et al., 2014, China [34]	*In vivo*	—	Linalool	—	*Pasteurella multocida* intranasal	Inflammation induced by biological agent

Jeena et al., 2014, India [35]	*In vivo*	Essential oil of *Piper nigrum* Linn	Caryophyllene	Piperaceae	Formalin	Inflammation induced by chemical agent

Entok et al., 2014, Turkey [36]	*In vivo*	Essential oil of *Nigella sativa* L.	—	Ranunculaceae	LPS from *Escherichia coli*	Inflammation induced by biological agent

Kazemi 2015, Iran [37]	*In vitro*	Essential oils *of Achillea millefolium* L., *Anethum graveolens* L., and *Carum copticum* L.	Thymol	Asteraceae Apiaceae	LPS from *Escherichia coli*	Inflammation induced by biological agent

Pinheiro et al., 2015, Brazil [38]	*In vivo*	Essential oil from leaves of *Choisya ternata* Kunth	—	Rutaceae	Dorsal subcutaneous injection of sterile air and carrageenan suspension	Inflammation induced by chemical agent

Kara et al. 2015, Turkey [39]	*In vivo*	—	Carvacrol	—	LPS from *Escherichia coli*	Inflammation induced by biological agent

Allam et al. 2015, Egypt [40]	*In vivo*	Essential oil of thyme	—	Lamiaceae	*Shigella flexneri*	Inflammation induced by biological agent

Shen et al. 2016, China [41]	*In vitro*	Essential oil of calyx of *Hibiscus sabdariffa L.*	*n*-Hexadecanoic acid	Malvaceae	LPS from *Escherichia coli*	Inflammation induced by biological agent

Park et al., 2016, Korea [42]	*In vitro and in vivo*	Essential oil of *Chamaecyparis obtusa*	—	Cupressaceae	*In vitro*: LPS from *Escherichia coli* *In vivo:* carrageenan-induced paw edema, thioglycollate-induced peritonitis	Inflammation induced by biological and chemical agent

Skala et al., 2016, Poland [43]	*In vitro*	Essential oils from roots of *Rhaponticum carthamoides*	Cyperene Aplotaxene	Asteraceae	LPS from *Escherichia coli*	Inflammation induced by biological agent

Zhao et al., 2016, China [44]	*In vivo*	—	Cinnamaldehyde	—	LPS from *Escherichia coli*	Inflammation induced by biological agent

Yu et al., 2016, Brazil [45]	*In vivo*	—	Thymol	—	High-fat-diet-induced hyperlipidemia and atherosclerosis.	Inflammation induced by chemical agent

Kennedy-Feitosa et al. 2016, Brazil [46]	*In vivo*	—	Eucalyptol	—	Exposition to commercial cigarettes	Inflammation induced by chemical agent

Alvarenga et al. 2016, Brazil [47]	*In vivo*	—	Carvacrol	—	Irinotecan	Inflammation induced by chemical agent

Shen et al., 2017, China [48]	*In vitro*	Essential oil from blossoms of *Citrus aurantium*	—	Rutaceae	LPS from *Escherichia coli*	Inflammation induced by biological agent

Liu et al., 2017, China [49]	*In vivo*	—	*β*-Elemene	—	High-fat-diet-induced hyperlipidemia and atherosclerosis	Inflammation induced by chemical agent

Leelarungrayub et al. 2017, Thailand [50]	*In vivo*	Essential oil of *Zingiber cassumunar* Roxb. in niosomes entrapped	Terpinen-4-ol	Zingiberaceae	LPS from *Porphyromonas gingivalis*	Inflammation induced by biological agent

Arigesavan and Sudhandiran 2017, India [51]	*In vivo*	—	Carvacrol	—	1,2-Dimethylhydrazine (DMH) and dextran sodium sulphate (DSS)	Inflammation induced by chemical agent

Marques et al., 2018, Brazil [52]	*In vitro*	—	l-Carveol, l-carvone, *m*-cymene, valencene, and guaiene	—	LPS from *Escherichia coli*	Inflammation induced by biological agent

Pivetta et al. 2018, Brazil [53]	*In vivo*	—	Thymol in nanoparticles from natural lipids	—	Imiquimod	Inflammation induced by chemical agent

**Table 2:** *In vitro* studies involving essential oils, anti-inflammatory and antioxidant activity.

Essential oil and/or majority constituent	Doses	Antioxidant and anti-inflammatory assays	Cell line	General results and proposed mechanism of action	Reference
Essential oils of the aerial part of *Eucalyptus bridgesiana*, *Cymbopogon martinii*, *Thymus vulgaris*, *Lindernia anagallis*, and *Pelargonium fragrans*	0.01 *μ*g/mL	*β*-Carotene linoleic acid bleaching test, DPPH radical, and nitric oxide scavenging assay 5-LOX inhibition assay Measurement of IL-1*β*, IL-8, TNF-*α*	THP-1 (human mylomonocytic cell)	Strong antioxidant activity in the tests performed; inhibition of 5-LOX activity and reduction of IL-1*β*, IL-8, and TNF-*α* secretion in THP-1 cells	Tsai et al. 2011 [24]

Essential oils of the aerial parts of *Oenanthe crocata L.*, *β-*ocimene, or sabinene	EO: 0.08, 0.16, and 0.32 *μ*L/mL *β-*Ocimene and sabinene: 0.32–1.25 *μ*L/mL	Measurement of NO, Western blot analysis for iNOS, and nitric oxide scavenging activity	RAW 264.7 macrophages	Strong NO scavenging activity and inhibition of iNOS expression Sabinene exhibited NO scavenging activity only at higher concentrations	Valente et al. 2013 [28]

Essential oils from leaves and stems of *Liquidambar Styraciflua*	1, 10, 100 and 500 *μ*g/mL	5-LOX and PGE_2_ inhibition DPPH radical and superoxide scavenging activity	HepG-2 cells	Reduction of DPPH, (OH^•^), and (O_2_^•^) radicals Inhibition of 5-LOX and PGE_2_	El-Readi et al. 2013 [27]

Essential oil of *Patrinia scabiosaefolia*	50, 100, 150, 200, and 250 *μ*g/mL	Measurement of IL-1 and IL-6 DPPH radical scavenging assay	BV-2 cell (microglia)	Inhibition of the production of IL-1 and IL-6; scavenging activity against the DPPH radical	Jing et al. 2014 [29]

Essential oil from leaf and branches of *Ocotea quixos*, wood, and branches and leaves of *Callitris intratropica* and *Copaifera reticulata/langsdorffii* gum-resin	5, 10, an 20 *μ*g/mL	*β*-Carotene linoleic acid bleaching test and DPPH radical scavenging assay Measurement of NO production Western blotting analyses for the iNOS and COX-2 and measurement of IL-8, IL-6, and IL-1*β*	RAW 264.7 macrophages	The EO of *O. quixos* and *C. reticulata* did not possess an antioxidant activity, while Blue Cypress possessed a moderate antioxidant activity Only Ocotea suppress the LPS-induced PGE_2_ production, LPS-mediated iNOS, and COX-2 elevation Suppression of LPS-stimulated IL-8 and IL-1*β* production in the cells	Destryana et al. 2014 [30]

Essential oils *of Achillea millefolium* L., *Anethum graveolens* L., and *Carum copticum* L.		DPPH radical scavenging and FRAP assay *β*-Carotene bleaching test Determination of NO production.	RAW 264.7 macrophages	*A. millefolium* had the highest antioxidant activity in all conducted assays and inhibited nitric oxide production	Kazemi 2015, Iran [37]

Essential oil of calyx of *Hibiscus sabdariffa L.*	25, 50, 100, 200, and 300 *μ*g/mL	Determination of NO production Measurement of cytokines Production (IL-1 and IL-6) RT-PCR assay of IL-1, IL-6, TNF-*α*, iNOS, and COX-2 mRNA Western blot analyses for the p-JNK, p-ERK1/2, NF-*κ*B, and GAPDH	RAW 264.7 macrophages	Inhibition of NF-*κ*B signaling pathways and MAPK (JNK and ERK1/2), reduction of NO production and IL-1, IL-6, TNF-*α*, COX-2, and iNOS	Shen et al. 2016 [41]

Essential oil of *Chamaecyparis obtusa*	*In vitro*: 1, 10, 50 and 100 *μ*g/mL *In vivo*: 5 and 10 mg/kg	*In vitro:* measurement of NO, IL-1*β*, TNF-*α*, and IL-6 by levels; Western blot analyses for expression of iNOS and COX-2 *In vivo:* carrageenan-induced paw edema and thioglycollate-induced peritonitis	RAW 264.7 macrophages	Decreasing in the number of total cells and suppression of TNF-*α*, IL-1*β*, and IL-6 levels in peritoneal fluid Suppression of iNOS and COX-2 expression	Park et al. 2016 [42]

Essential oils from roots of *Rhaponticum carthamoides*	25, 50, and 100 *μ*g/mL	Measurement of cytokines IL-1*β*, IL-6, IL-8, IL-10, TNF-*α*, and GM-CSF and RT-PCR. ROS formation assay using H2DCF-DA.	Human astrocytes	Decreasing the expression of IL-1*β*, IL-6, and TNF-*α* and the ROS level	Skala et al. 2016 [43]

Essential oil from blossoms of *Citrus aurantium*	15.625, 31.25, 62.5, 125, and 250 *μ*g/mL	DPPH and ABTS radical scavenging activity Determination of morphology and NO production. Quantification of IL-6, TNF-*α*, and IL-1*β* Reverse transcription and PCR-RT for iNOS	RAW 264.7 macrophages	Did not show scavenging effects on DPPH and ABTS radicals Inhibition of NO accumulation and suppression of IL-6, TNF-*α*, and IL-1*β* secretion Inhibition of the expression of COX-2 and NF-*κ*B activation Involvement of the inflammation process through MAPK signaling pathways, by inhibiting phosphorylation of JNK (c-Jun N-terminal kinase) and p38	Shen et al. 2017 [48]

l-Carveol, l-carvone, *m*-cymene, valencene, and guaiene	1, 10, and 100 *μ*M	Protective effect against oxidative damage produced by superoxide anion production (O_2_^·−)^ and hydrogen peroxide Determination of NO production Quantification of IL-1*α*, TNF-*α*, and IL-10 Activity of NF-*κ*B	RAW 264.7 macrophages	Reduction in TNF-*α* and IL-1*α* levels and increasing in the production of IL-10 Guaiene and *m*-cymene inhibited NO production l-Carveol and *m*-cymene significantly inhibited O_2_^·−^ production Terpenes suppressed NF-*κ*B activity	Marques et al. 2018 [52]

EO: essential oil; NO: nitric oxide; ROS: reactive oxygen species; iNOS: inducible nitric oxide synthase; IL-1*β*: interleukin-1 beta; DPPH: 2,2-diphenyl-1-picrylhydrazyl radical; LPS: lipopolysaccharide; M: male; ABTS: 2,2′-azino-bis(3-ethylbenzothiazoline-6-sulphonic acid); NF-*κ*B: nuclear factor kappa B, COX-2: cyclooxygenase; TNF-*α*: tumor necrosis factor alpha; IL-6: interleukin-6; IL-8: interleukin-8; IL-10: interleukin-10; FRAP: ferric reducing antioxidant power; MPO: myeloperoxidase; PCR-RT: real-time quantitative polymerase chain reaction; H2DCF-DA: 2′,7′-dichlorodihydrofluorescein diacetate; 5-LOX: 5, lipoxygenase; PGE2: prostaglandin E2; MAPK: mitogen-activated protein kinases; GAPDH: glyceraldehyde 3-phosphate dehydrogenase.

**Table 3:** *In vivo* studies involving essential oils and antioxidant activity.

Essential oil and/or majority constituent	Animals (strain/sex), *n* (per group)	Doses, route, and administration period	Antioxidant assays	Experimental model of inflammation	General results	Reference
Essential oil of ginger	Mice (Balb/c/), *n* = 3, 5, or 6	10, 50, 100, 250, 500, or 1000 mg/kg (i.p. or p.o.), single dose or 4 days	Lipid peroxidation, SOD and hydroxyl activity assay DPPH and ABTS radical scavenging and FRAP assay PMA-induced radical generation and dosage of SOD, CAT, and GSH *in vivo*	Formalin induced chronic inflammation	Scavenged superoxide, DPPH, hydroxyl radicals, and lipid peroxidation inhibition Increase in SOD, GSH, and GR enzyme levels in blood and glutathione peroxidase and SOD enzymes in liver Reduction in formalin-induced chronic inflammation	Jeena et al. 2013 [26]

Anethole	Mice (Swiss/M), *n* = 6	125, 250, or 500 mg/kg (p.o.), for until 7 days	MPO activity	Paw edema induced by complete Freund's adjuvant	Inhibition of paw edema on all of the days analyzed Inhibition of MPO activity and reduction of TNF-*α*, IL-1*β*, and IL-17 levels in acute and persistent inflammation models	Ritter et al. 2013 [25]

Essential oil of *Nigella sativa* L.	Rats (Sprague–Dawley), *n* = 7	500 mg/kg (p.o.), 3 times a 1 day	Determination of SOD, CAT activity, and MDA and NO levels	LPS induced inflammation	Increase in SOD and CAT, and reduction of MDA and NO in lung	Entok et al. 2014 [36]

Essential oil of *Piper nigrum* Linn	Mice (Balb/C), *n* = 5 or 6	10, 50, 100, 250, 500, or 1000 mg/kg (i.p. or p.o.), 5 or 30 days.	Lipid peroxidation and SOD and hydroxyl activity assay DPPH radical scavenging and FRAP assay PMA-induced radical generation and dosage of SOD, CAT, and GSH *in vivo*	Formalin induced chronic inflammation	Scavenged SOD, DPPH, and hydroxyl radicals; inhibition of lipid peroxidation *in vitro* Increase in SOD and GSH enzyme levels in blood of mice and CAT, SOD, and GSH enzymes in liver Reduction of chronic inflammation in formalin test	Jeena et al., 2014 [35]

Linalool	Mice (C57BL/6J/M), *n* = 10	5, 15 or 25 mg/kg (s.c.)	ROS and SOD activity assay	*Pasteurella multocida* induced intranasal lung infection/inflammation	Increase in nuclear Nrf-2 protein amount and reduction in SOD expression Reduction in TNF-*α* and IL-6 levels and decrease in neutrophil accumulation	Wu et al. 2014 [34]

Essential oil from blossoms of *Citrus aurantium* L.	Rats (Wistar/M), *n* = 8	5, 10, 20, 40, or 80 mg/kg (i.p.) for until 7 days	Measurement of NO	Cotton pellet-induced granuloma	Decrease in transudate and granuloma formation amount involving the nitric oxide pathway	Khodabakhsh et al. 2014 [33]

Essential oil of *Camellia reticulata* L.	Rats (Wistar/M), *n* = 6	200 or 400 mg/kg (p.o.) for 11 days	Colonic GSH content and lipid peroxides concentration	Enterocolitis induced by indomethacin	Decrease in macroscopic and microscopic scores for inflammation Reduction in MPO and lipid peroxidation and increase in GSH content	Patil et al., 2014 [32]

Essential oil of *Pistacia integerrima*	Rats (Sprague-Dawley/F), *n* = 6	5-30 *μ*g/mL; 10, 30, or 100 mg/mL; and 7, 5, 15, or 30 mg/kg (i.p.)	DPPH radical scavenging, lipoxygenase activity, and measurement of NO and MPO	LPS- and ovalbumin-induced bronchial inflammation	Inhibition of lipoxygenase enzyme and DPPH scavenging activity Antiallergic activity by inhibition of mast cell degranulation Reduction in total leucocyte, neutrophils, NO, total protein, and albumin levels in bronchoalveolar fluid and MPO levels in lung homogenates	Shirole et al. 2014 [31]

Essential oil of thyme	Rats (Sprague-Dawley/M), *n* = 25	7, 5, 15, or 30 mg/kg (i.p.) for 21 days	FRAP assay	Ulcer-forming induced by *Shigella flexneri strain*	Synergistic activity of thyme oil decreased the inflammation of the lamina propria and decreased the bacterial load in the colon Increase in total antioxidant capacity time	Allam et al. 2015 [40]

Essential oil from leaves of *Choisya ternata* Kunth	Mice (Webster/M), *n* = 4, 6, 8 or 10	3-10 or 30 mg/kg (p.o.)	NO levels and trapping capacity of anthranilates	Formalin test and subcutaneous air pouch (SAP) model	Reduction in migration, exudate volume, and protein extravasation and reduced levels of NO, TNF-*α*, and IL-1*β*	Lin et al. 2014 [29]

Carvacrol	Rats (Sprague-Dawley/F), *n* = 6, 7, or 8	20, 40, or 80 mg/kg (p.o.) for 6 days	MDA and NO levels	LPS-induced peritoneal inflammation	Decrease in levels of TNF-*α* and IL-6, MDA, NO levels, and arginase activity levels	Kara et al., 2015 [39]

Cinnamaldehyde	Rats (Sprague-Dawley/M), *n* = 6	30, 60, or 90 (p.o.) 1x/day for 30 days	Determination of intracellular levels of ROS	LPS-induced cardiac dysfunction	Inhibition of cardiac dysfunction, inflammatory infiltration, and the levels of TNF-*α*, IL-1*β*, and IL-6 in LPS stimulated rats by blocking the TLR4, NOX4, MAPK, and autophagy signaling pathway	Zhao et al., 2016 [44]

Thymol	Rabbits (M), *n* = 6	3 or 6 mg/kg (p.o) for 8 weeks	DPPH and ABTS radical scavenging activity and measurement of MDA level in serum	Inflammatory process in aortic intimal thickening	High antioxidant activity in both tests Reduction in TC, TG, LDL-C, and MDA levels Reduction in VCAM-1 and MCP-1 levels and proinflammatory cytokines IL-1*β*, IL-6, and TNF-*α*	Yu et al. 2016 [45]

Eucalyptol	Mice (C57BL/6/M), *n* = 8	1, 3, and 10 mg/mL via inhalation (15 min/daily) for 5 days	NBT assay, SOD and CAT activity Measurement of GSH and TBARS levels	Cigarette smoke exposure	Reduction in IL-1*β*, IL-6, and TNF-*α* levels Decrease in NF-*κ*B expression Reduction in ROS, SOD, CAT, MDA, and GSH levels Rare presence of leukocytes in alveolar septa	Kennedy-Feitosa et al., 2016 [46]

Carvacrol	Mice (Swiss/F), *n* = 5 or 8	25, 75, or 150 mg/kg (i.p.) for 8 days	GSH, MDA, and NO levels	Intestinal mucositis induced by CPT-11 chemotherapy	Reduction in TNF-*α*, IL-1*β*, and KC levels Decrease in MPO, NF-*κ*B, COX-2, and oxidative stress (GSH, MDA, and NO levels)	Alvarenga et al. 2016 [47]

*β*-Elemene	Mice ApoE−/− (C57BL/6/M), *n* = 6	Not related	Measurement of eNOS and NO concentrations, ROS assay, enzyme activity SOD, CAT, GPx, GSH, and MDA	Atherosclerosis induced by high fat	Inhibition of atherosclerotic lesion size and increase in plaque stability Reduction of vascular oxidative stress and preventing proinflammatory cytokine production Improvement in NO levels, expression of eNOS, and phosphorylation of eNOSser1177 and Akt	Liu et al. 2017 [49]

Carvacrol	Rat (Fischer 344/M), *n* = 6	50 mg/kg (p.o.) for 7 days before and 7 days, after tumor induction	Antioxidant enzyme activities SOD, CAT, GPx, GR, GSH, vitamin E and vitamin C, and NO level and MDA contents	Colitis induced by DMH-associated colon cancer	Increase in SOD, CAT, and GSH levels and reduction in LPO, MPO, and NO Suppression of proinflammatory mediators iNOS and IL-1*β* Reduction in ulcer size	Arigesavan and Sudhandiran 2017 [51]

Essential oil of *Zingiber cassumunar* Roxb. in niosomes entrapped	Rats (Wistar/M), *n* = 5	12.5–400 *μ*g/mL	DPPH radical scavenging	LPS-induced subcutaneous inflammatory assay	Inhibition of DPPH radical and decrease in skin temperature and blood flow, reducing tissue inflammation process	Leelarungrayub et al. 2017 [50]

Thymol in nanoparticles from natural lipids	Mice (C57B/6/M), *n* = 10 or 12	5 mg/day (p.o.), 15 days	Anthralin-induced ear edema model	Imiquimod-induced psoriasis	Improved inflammation and healing, on anthralin model and imiquimod	Pivetta et al. 2018 [53]

eNOS: nitric oxide synthase; NO: nitric oxide; ROS: reactive oxygen species; SOD: superoxide dismutase; CAT: catalase; GPx: glutathione peroxidase; GSH: glutathione; GR: reductase glutathione; MDA: malondialdehyde; DMH: 1,2-dimethyl hydrazine; LPO: lipid peroxides; iNOS: inducible nitric oxide synthase; IL-1*β*: interleukin-1 beta; DPPH: 2,2-diphenyl-1-picrylhydrazyl radical; LPS: lipopolysaccharide; M: male; ABTS: 2,2′-azino-bis(3-ethylbenzothiazoline-6-sulphonic acid); TC: total cholesterol; TG: triglycerides; LDL: low-density lipoprotein; VCAM-1: vascular cell adhesion molecule-1; MCP-1: monocyte chemotactic protein-1 (MCP-1); NBT: nitroblue tetrazolium; TBARS: thiobarbituric acid; NF-*κ*B: nuclear factor kappa B; MDA: malondialdehyde; CPT-11: irinotecan, Camptosar, Camptothecin-11; COX-2: cyclooxygenase; TNF-*α*: tumor necrosis factor alpha; IL-6: interleukin-6; IL-17: interleukin-17; FRAP: ferric reducing antioxidant power; MPO: myeloperoxidase; TNBS: trinitrobenzenesulphonic acid; Nrf-2: nuclear factor erythroid 2–related factor 2; PMA: phorbol-12-myristate-13-acetate.
